# Huntington disease exacerbates action impulses

**DOI:** 10.3389/fpsyg.2023.1186465

**Published:** 2023-06-16

**Authors:** Shuhei Shiino, Nelleke Corine van Wouwe, Scott A. Wylie, Daniel O. Claassen, Katherine E. McDonell

**Affiliations:** ^1^Department of Neurology, Vanderbilt University Medical Center, Nashville, TN, United States; ^2^Department of Neurological Surgery, University of Louisville, Louisville, KY, United States

**Keywords:** Huntington disease, interference, inhibitory control, motor impulsivity, Simon task

## Abstract

**Background:**

Impulsivity is a common clinical feature of Huntington disease (HD), but the underlying cognitive dynamics of impulse control in this population have not been well-studied.

**Objective:**

To investigate the temporal dynamics of action impulse control in HD patients using an inhibitory action control task.

**Methods:**

Sixteen motor manifest HD patients and seventeen age-matched healthy controls (HC) completed the action control task. We applied the activation-suppression theoretical model and distributional analytic techniques to differentiate the strength of fast impulses from their top-down suppression.

**Results:**

Overall, HD patients produced slower and less accurate reactions than HCs. HD patients also exhibited an exacerbated interference effect, as evidenced by a greater slowing of RT on non-corresponding compared to corresponding trials. HD patients made more fast, impulsive errors than HC, evidenced by significantly lower accuracy on their fastest reaction time trials. The slope reduction of interference effects as reactions slowed was similar between HD and controls, indicating preserved impulse suppression.

**Conclusion:**

Our results indicate that patients with HD show a greater susceptibility to act rapidly on incorrect motor impulses but preserved proficiency of top-down suppression. Further research is needed to determine how these findings relate to clinical behavioral symptoms.

## Introduction

Huntington disease (HD) is a neurodegenerative disorder that results from an expanded cytosine-adenine-guanine (CAG) repeat in the HTT gene on chromosome 4. Worldwide, HD affects approximately 3 in 100,000 people, with the highest prevalence in North America, Europe, and Australia (Pringsheim et al., 2012). In the United States, an estimated 40,000 people carry a diagnosis of HD and over 200,000 are at risk (Huntington’s Disease Society of America, 2022). While HD has traditionally been diagnosed based upon the presence of motor abnormalities, data from longitudinal studies demonstrate that cognitive and behavioral symptoms begin decades before the onset of chorea (Paulsen et al., 2008; Tabrizi et al., 2009; Epping et al., 2016) and have even more detrimental effects on both patients and caregivers (Eddy et al., 2016). The hallmark of cognitive impairment in HD is frontal-executive dysfunction, which can take a variety of forms including slowed processing, deficits in attention and working memory, and impaired task-switching ability (Dumas et al., 2013; Tabrizi et al., 2013; Papoutsi et al., 2014; Eddy et al., 2016; Snowden, 2017; Migliore et al., 2019). Impairments in impulse control have also been described in HD, which can clinically manifest as impaired forethought and engagement in high-risk behavior (Jensen et al., 1998; Kalkhoven et al., 2014; van Wouwe et al., 2016a; Risk-taking behaviors in Huntington’s Disease – IOS Press, 2022). Previous studies have suggested that clinically observed impulsivity in HD may be linked to altered responsiveness to reward and deficits in inhibitory action control (Kalkhoven et al., 2014; Galvez et al., 2017; Johnson et al., 2017). However, the underlying cognitive dynamics of these behavioral manifestations remain unclear.

Neuropathologically, HD causes progressive degeneration of neostriatal medium spiny neurons and disruption to frontal-striatal circuitries (Wolf et al., 2008; Beste et al., 2010; Poudel et al., 2015; Wiecki et al., 2016; Soloveva et al., 2020). Alterations in the frontal-striatal network have been linked to impairments in inhibitory control and the development of impulsive-compulsive behaviors in other conditions such as Parkinson disease (Wylie et al., 2009a; van Wouwe et al., 2014) but have not yet been fully investigated in HD. A better understanding of the inhibitory action control processes that underlie clinically observed impulsivity in HD could help improve management of these challenging symptoms and contribute to the development of novel interventions. This is particularly critical given the burden of cognitive and behavioral symptoms in HD and the lack of evidence-based psychological interventions in this population (Zarotti et al., 2020).

**Figure 1:**
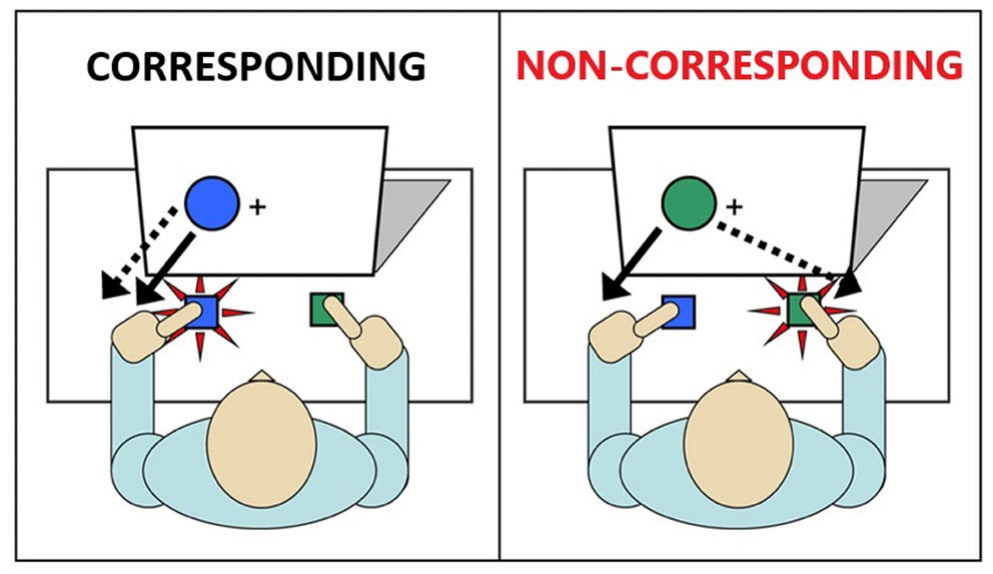
Illustration of the Simon task. Image on the left depicts a single trial in which the irrelevant stimulus and the goal-related stimulus are not conflicted (Corresponding). Image on the right depicts a single trial in which the irrelevant stimulus and the goal-related stimulus are conflicting (Non-corresponding).

One well-validated cognitive paradigm that simply and effectively assesses inhibitory action control is the Simon task (Simon, 1969). In this task, participants are instructed to respond with a left or right button press according to the color of a stimulus (e.g., green circle = right-thumb press; blue circle = left-thumb press) (Figure 1). The stimuli are presented either to the left or right of a central fixation point. While the spatial position of the stimulus is irrelevant to the participant’s goal, it nonetheless impacts performance by eliciting a spontaneous impulse to respond with the hand on the same side (i.e., a stimulus appearing in the left spatial half-field activates a left hand response impulse). When the response impulse activated by the spatial location of the stimulus is the same as the response signaled by the stimulus color (corresponding trials), reactions to the stimulus color are faster and more accurate compared to reactions to the stimulus color that appears in the opposite visual half-field (non-corresponding trials). That is, when the impulsive response signaled by the spatial location conflicts with the response signaled by the color, reaction times (RT) are slowed and response errors increase as participants act on the spatially-driven impulse. This decrement in performance on non-corresponding trials compared to corresponding trials is known as the Simon effect (Zorzi and Umiltà, 1995).

The advantage of the Simon task is that it elegantly distinguishes between two distinct aspects of impulse control: the strength of the initial response impulse (impulse capture) and the proficiency of a reactive inhibition mechanism to suppress the impulse (impulse suppression). The Dual Process Activation-Suppression (DPAS) model provides a theoretical and analytical framework to quantify these two processes by analyzing the full spectrum of the RT distribution (Kornblum et al., 1990; Kornblum, 1994). Impulse capture reflects the initial activation of motor impulses triggered by the stimulus location. This is visualized by plotting accuracy against RT (i.e., a conditional accuracy function, CAF) for each level of correspondence. On corresponding trials, accuracy rates are uniformly high across the entire distribution of RTs. In contrast, the CAF for non-corresponding trials reveal a marked increase in fast impulsive errors, the magnitude of which offers quantification of the strength of initial action impulses. In contrast, the process of suppressing activated impulses takes time to build up. This suppression process is visualized by plotting the Simon interference effect on RT (i.e., the cost to RT on non-corresponding trials relative to corresponding trials) across the RT distribution (i.e., a delta plot), which reveals a reduction in interference at the slow end of the distribution. The magnitude of the slope reduction of interference quantifies the proficiency of suppression, with larger negative-going slopes indicating more effective inhibition of impulses (De Jong et al., 1994; Ridderinkhof, 2002).

Previous studies have shown that HD patients respond more slowly and make more errors than HCs on the Simon task (Georgiou-Karistianis et al., 2007; Thiruvady et al., 2007). However, these studies are limited in number [only four citations in total (Cope et al., 1996; Georgiou-Karistianis et al., 2007; Thiruvady et al., 2007; Palmer et al., 2017)] and have not applied the DPAS model to distinguish between impulse capture and impulse suppression in the interpretation of these results.

In the current study, we utilized the Simon Task as an experimental framework to investigate the temporal dynamics of inhibitory control in HD patients and to test two competing hypotheses. First, if HD leads to stronger activation of motor impulses, we would expect to see differences in the CAF. Alternatively, if HD instead disrupts the ability to suppress motor impulses but does not alter the strength of these impulses, this would be reflected in the delta plot. Our study paradigm thus allows us to test these distinct hypotheses and determine whether impulsivity in HD may reflect impulse capture vs. impulse suppression.

## Materials and methods

### Participants

HD participants (*n* = 16) were recruited from the Huntington’s disease specialty clinic at Vanderbilt University Medical Center (VUMC). Age-matched HCs (*n* = 17) were recruited through community advertising. Exclusion criteria for all participants (HD and HC) included a history of other neurological conditions, unstable mood disorder, bipolar affective disorder, schizophrenia, other significant psychiatric or medical conditions that may compromise executive function, or a Montreal Cognitive Assessment (MoCA) score < 20 (Nasreddine et al., 2005). The MoCA was administered at a screening visit to both HD and HC participants by a trained research assistant. HD participants were diagnosed by a movement disorder neurologist and confirmed via genetic testing. Each of the HD participants met criteria for motor manifest HD of mild to moderate severity based on the Unified Huntington Disease Rating Scale (UHDRS). All participants had normal or corrected to normal vision. They provided written informed consent before participating in the study. Participation was voluntary and subjects received no compensation. The study was compliant with the standards of ethical conduct in human research and was approved by the Vanderbilt University Institutional Review Board (IRB #121810).

### Experimental procedures

Participants were seated in a comfortable armchair in a separate study room. The Simon task was administered on a desktop computer running Windows operating system connected to a 17-inch monitor located approximately 1 meter from participants’ eye level. The task began with presentation of a light gray screen background and a black fixation point (small square) located at the center of the gray screen; the fixation point remained on the screen for the duration of the task. Participants were instructed to focus on the fixation point and to be ready to respond as quickly and as accurately as possible to the appearance of circle that would appear to the left or right of the fixation point along the horizontal plane. Within a variable duration of 1750–2,250 ms after the onset of the fixation point, a blue or green circle (diameter 2.1 cm; visual angle 1.20°) appeared to the left or to the right of the fixation point with an edge-to-edge separation of 0.6 cm between the circle and fixation point. Participants held a response device in each hand with a button fixed at the top of the device allowing for comfortable button presses with the thumb of each hand. They were asked to respond by pressing the left or right button based on a pre-instructed mapping between the color of the stimulus and a response (e.g., green circle = right button press; blue circle = left button press). The assignment of green and blue to left or right hands was counterbalanced across participants. The circle stimulus remained on the screen until the participant made a response or 1,500 ms elapsed, upon which the circle disappeared. A variable intertrial interval (1,750–2,250 ms in increments of 50 ms) then transpired before another blue or green circle appeared to either side of the fixation point, thus signaling the start of the next trial. Participants completed 60 stimulus trials per block, first completing a practice block of 60 trials followed by 4 experimental blocks (240 total experimental trials). Participants were provided a brief break between blocks. For a more detailed description of the task, see van Wouwe et al. (2016b).

Trials were defined as corresponding (Cs) if the circle appeared on the same side as the assigned response (e.g., a blue circle calling for a left response appeared to the left side of fixation). Trials were defined as non-corresponding (Nc) if the circle appeared on the opposite side of the assigned response (e.g., a blue circle calling for a left response appeared to the right side of fixation). The color-response mapping was counterbalanced across participants, and Cs and Nc trials were presented randomly but with equal probability across hands.

### Data analysis

Reaction time on each trial was computed from the onset of the circle stimulus to the button press. Mean RT and accuracy rates were analyzed using separate repeated-measures ANOVAs with a within-subjects factor of *Correspondence* (corresponding and non-corresponding) and a between-subjects factor of *Group* (HD and HC). Alpha level was set at 0.05 for all analyses. For each participant, RT values on any single trial that were faster than 180 ms (anticipatory responses) or slower than three standard deviations above or below their mean were identified as outliers and excluded (van den Wildenberg et al., 2010). This accounted for fewer than 1% of trials across participants. Two participants (one HD patient and one HC) were found to have accuracy rates more than two standard deviations below the mean on both Cs and Nc trials and were thus excluded from the analyses.

We used the previously described DPAS model to differentiate between impulse capture and impulse suppression. To compute impulse capture, we first rank-ordered RTs from all trials (correct and incorrect) separately for Cs and Nc trials, and then binned these distributions into 6 equal sized bins (i.e., equal numbers of trials in each bin). Next, we computed accuracy rates for each bin and plotted those values against the mean RT for that bin, producing a conditional accuracy function for Cs and for Nc trial types. This visualized accuracy rates as a function of reaction speed. The accuracy rate from the fastest bin of trials for Nc trial types provides a measure of the strength of impulse capture, with lower accuracy rates indicating a stronger susceptibility to committing fast impulsive reaction errors (i.e., stronger impulse capture). We compared HD and HC groups on this measure of impulse capture using a repeated measures ANOVA. For impulse suppression, we first rank-ordered all correct response trials separately for Cs and Nc trial types, and then binned these distributions into 6 equal sized bins. For each bin, we computed a Simon effect (mean Nc RT minus mean Cs RT) and plotted this delta value against the average RT of all trials in that bin (i.e., a delta plot). This visualized the interference effect across the distribution of RTs. Because inhibition takes time to build up, the suppression of the impulse is best measured by the slope reduction of interference between the final two points of the delta plot. The typical finding is a negative-going slope where the interference effect is reduced at slower reaction times, thus indicating suppression of interference. We compared the final slope between groups using a one-way ANOVA. For a more detailed description of the DPAS model and distributional analytical methods, see van den Wildenberg et al. (2010). These methods have been applied across empirical studies of clinical and nonclinical populations (Burle et al., 2002; Ridderinkhof et al., 2005; Bub et al., 2006; Wijnen and Ridderinkhof, 2007; Wylie et al., 2007, 2009a,b, 2010).

## Results

### Analysis of sample demographics

Demographic characteristics of HD patients and HCs are shown in Table 1. The groups were similar in age [*t*(31) = 0.35, *p* = 0.73] and gender distribution [*Χ*^2^(1, 33) = 0.24, *p* = 0.62]. HCs were more likely to be right-handed (*p* = 0.04) [*Χ*^2^(1, 33) = 4.25, *p* = 0.04]. And to have a higher level of education [*t*(31) = 3.84, *p* = 0.001] than HD participants. HD participants had a mean CAG repeat length of 42.4 (*SD* 6.5) and a CAG-Age-Product (CAP) score of 466.7 (*SD* 214.34) [CAP score = age × (CAG − 33.66)]. Mean UHDRS total motor score was 25.4 (*SD* 16.9). The mean number of years since symptom onset was 6.1 (*SD* 4.2) and the number of years since diagnosis was 5.0 (*SD* 4.8).

**Table 1:** Sample characteristics.

	HD patients	Controls	*p*-value
Sample size	16	17	
Age (years)	44.3 (14.0)	45.9 (12.9)	0.73
Gender (M:F)	6:10	5:12	0.62
Education (years)	13.6 (2.3)	16.7 (2.4)	0.001^*^
Handedness (L:R)	7:9	2:15	0.04^*^
CAG repeat length	42.4 (6.5)		
CAP score	366.7 (214.34)		
UHDRS total motor score	25.4 (16.9)		
Years since symptom onset	6.1 (4.2)		
Years since diagnosis	5.0 (4.8)		

Values are presented as mean (standard deviation). ^*^p < 0.05.

### Mean Simon interference effects

Table 2 shows mean RTs and accuracy rates on corresponding and non-corresponding trials by group. *F*-values for main effects and interaction effects are shown in Table 3. Overall, responses among HD participants were slower and less accurate (591.6 ms, 0.96 accuracy rate) compared to HC (460.0 ms, 0.99 accuracy rate) [Group, RT: *F*(1, 29) = 13.02, *p* < 0.01; Acc: *F*(1, 29) = 8.96, *p* < 0.01].

**Table 2:** Means and standard deviations for reaction time and accuracy on corresponding (Cs) and non-corresponding (Nc) trials in HD patients and healthy controls.

	Group	
	HD (*n* = 16)	HC (*n* = 17)
Cs RT (ms)	559.6 (113.2)	447.8 (81.9)
Nc (ms)	623.6 (128.1)	473.0 (79.1)
Cs accuracy (% correct)	98 (0.01)	99 (0.01)
Nc accuracy (% correct)	94 (0.06)	98 (0.03)

**Table 3:** *F*-values for main effects and interaction effects on Simon task variables.

	Accuracy	RT
Group	8.96^*^	13.02^*^
Correspondence	10.17^*^	89.96^**^
Group × correspondence	2.01	17.00^**^

^*^p < 0.01; ^**^p < 0.001.

Across all participants, mean response latencies were faster and accuracy was higher on Cs than Nc trials, producing the expected Simon effect [Correspondence, RT: *F*(1, 29) = 89.96, *p* < 0.001; Acc: *F*(1, 29) = 10.17, *p* < 0.01]. HD participants showed approximately 250% larger slowing on Nc compared to Cs trials (HD: 64 ms; HC: 25 ms), indicating an exacerbated interference effect on RT [Correspondence × Group, RT: *F*(1, 29) = 17.00, *p* < 0.001]. The magnitude of the reduction in accuracy on Nc compared to Cs trials was similar between HD patients and HCs (HD: 0.04; HC: 0.01) [Correspondence × Group, Accuracy: *F*(1, 29) = 2.01, *p* = 0.167].

**Figure 2:**
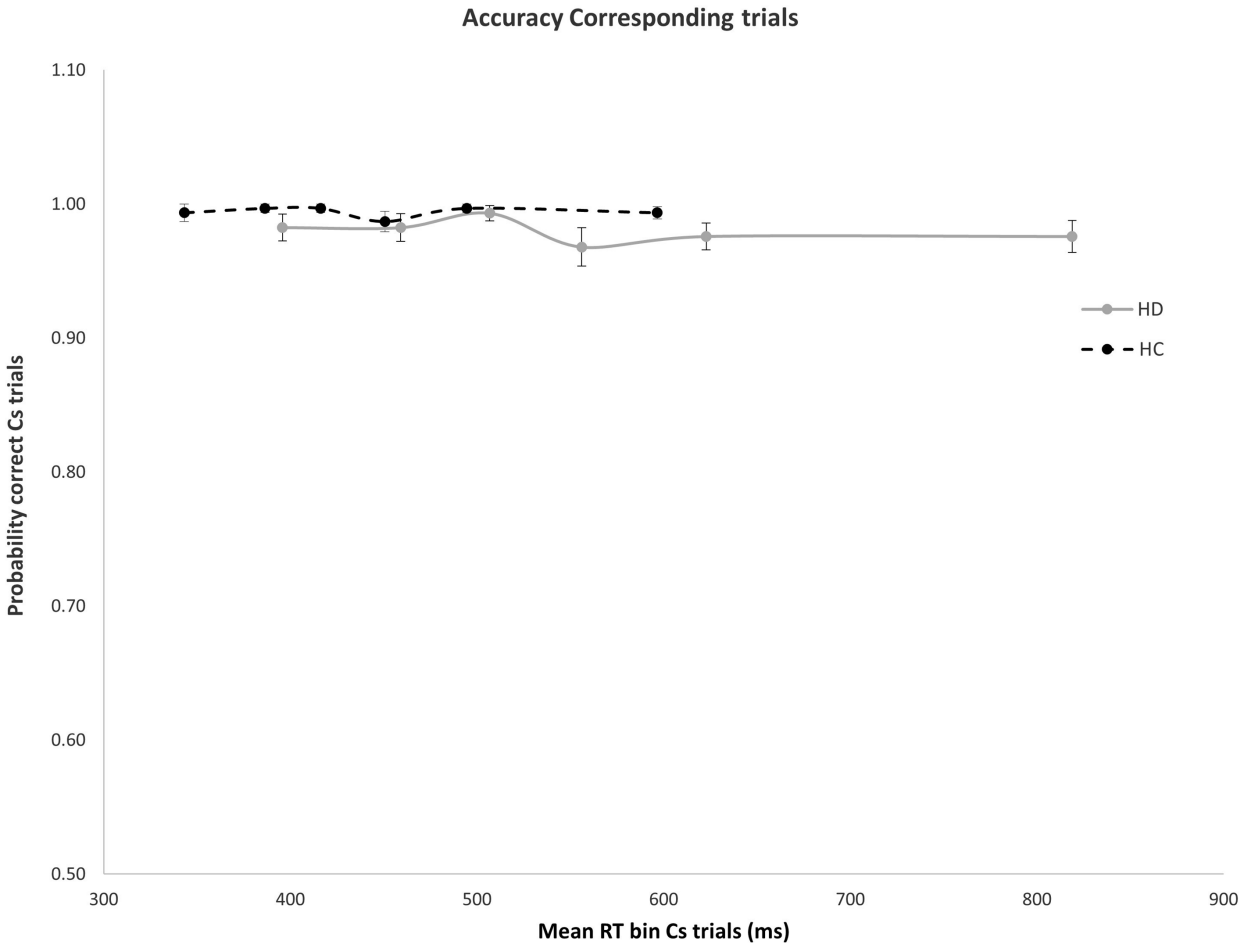
No significant difference was found in mean accuracy rates across RT bins for Cs trials in HD participants and HCs. Error bars reflect SEMS.

### Impulse capture

**Figure 3:**
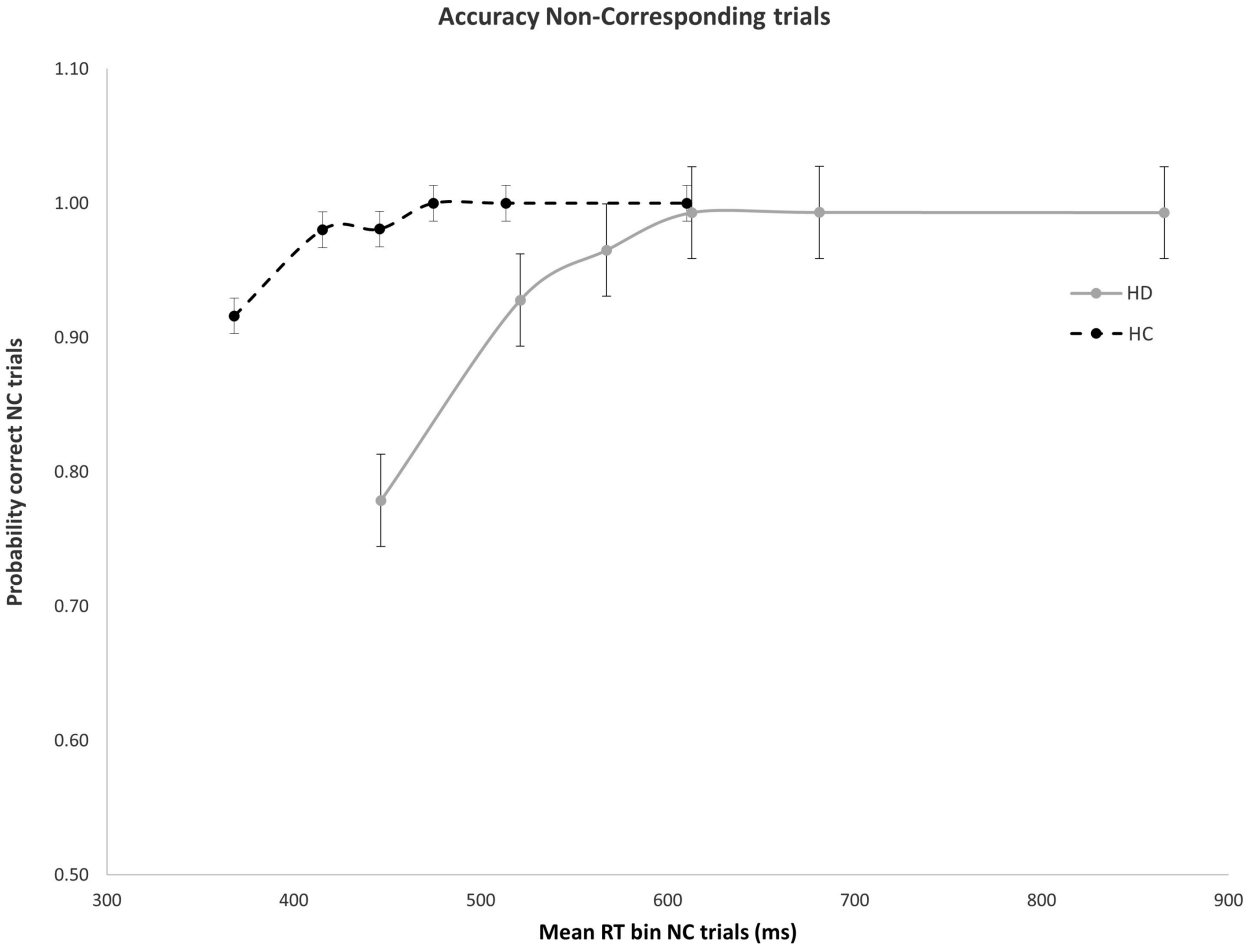
Mean accuracy rates across RT bins for Nc trials in HD participants and HCs. Percent accuracy is significantly diminished within the earliest bin in HD patients. Error bars reflect SEMs.

As expected, error rates were confined to the fastest portion of the conditional accuracy function plot. Moreover, fast errors occurred predominantly on Nc trials where the impulse to react to the side of the circle location captured the action system [Correspondence, Accuracy: *F*(1, 29) = 26.207, *p* < 0.001]. HD patients made significantly more fast errors than HC participants [Group, Accuracy: *F*(1, 29) = 6.658, *p* = 0.015], but this difference depended on the correspondence level [Correspondence × Group, Accuracy: *F*(1, 29) = 5.302, *p* = 0.029]. Specifically, whereas HD and HC groups made few and similar levels of fast errors on Cs trials (Figure 2), HD patients committed significantly more fast errors than HC on Nc trials where the spatial location of the circle produced a strong incorrect impulse [HD: 0.78; HC: 0.92; *F*(1, 29) = 6.193, *p* = 0.019] (Figure 3).

### Impulse suppression

**Figure 4:**
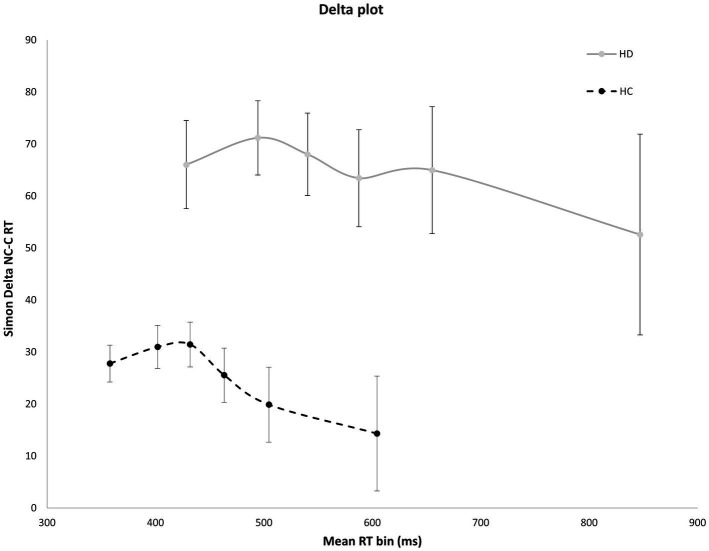
Mean interference effect (RT Nc minus Cs trials) across mean RT bins for HD and HC. The delta plot shows a similar reduction in interference (negative going delta slope) between last two RT bins for HD participants and HCs. Error bars reflect SEMs.

Figure 4 shows the interference effect across the entire delta plot function. As is typical, interference increases initially, then tapers and reverses at the slow end of the delta plot. Notably, the magnitude of interference is markedly larger across the entire plot for HD patients compared to HC. However, the slope reduction at the slow end of the delta plot is negative-going and similar in magnitude among HD and HC groups, suggesting that suppression was equally proficient among the groups. HD patients and HCs both show negative going delta plot slope values in the final two RT distribution bins [HD: −0.65; HC: −0.53, *F*(1, 29) = 0.14, *p* = 0.906], indicating that HD participants and HCs are equally proficient at inhibiting interference from action impulses (Figure 4).

## Discussion

Overall, participants with HD were slower to respond and less accurate than HCs on the Simon task. HD patients were significantly more susceptible to the Simon interference effect, showing a greater slowing of RT on trials with action conflict (Nc) compared to when there was no conflict (Cs). This is consistent with previous studies showing impaired inhibitory control on a variety of cognitive neuroscience tasks in HD patients, including the stop signal task (Aron et al., 2003; Verbruggen et al., 2008), Go/Nogo task (Beste et al., 2008), and Simon task (Georgiou-Karistianis et al., 2007; Thiruvady et al., 2007).

The distributional analyses included in this study provided novel insights into the dissociable effects of impulse capture and impulse suppression that were not evident in the mean results and have not been previously reported in HD. This analysis demonstrated that HD patients experience significantly stronger capture by the activation of response impulses, evidenced by an exacerbation of the number of fast, impulsive errors on Nc conflict trials. This stronger initial activation by prepotent response impulses was also evidenced by significantly higher interference costs at the early and late phases of the delta plot. In other words, the elicitation of strong reactive impulses produces a higher propensity for committing fast action errors as well as greater interference with the selection of a goal response. Importantly, higher rates of fast impulsive errors were not the result of a speed-accuracy trade-off as HD patients were both slower to respond and showed higher fast impulsive errors. In contrast, the counteractive mechanism that suppresses this interference, evidenced by the slope reduction of interference at the slow end of the delta plot, was similar among HD and HC groups. This dissociation reveals that HD primarily alters the way prepotent response impulses are activated as opposed to suppressed in the motor system.

Increased susceptibility to fast impulsive errors on the Simon task has been linked to increased activity in the pre-supplementary motor area (SMA) in functional imaging studies (Ullsperger and von Cramon, 2001; Cisek, 2007). Suppression of conflict, on the other hand, appears to rely on the engagement of fronto-parietal and fronto-striatal circuits responsible for top-down inhibitory action control (Williams-Gray et al., 2007; Ghahremani et al., 2012). Specifically, activation of the right inferior frontal cortex has been shown to be associated with greater negative-going delta slope values, representing more efficient conflict suppression (Forstmann et al., 2008). Based on these patterns, earlier stage HD may produce alterations in pre-SMA activity or pre-SMA-basal ganglia circuity involved during the initial action activation and selection, while leaving suppression mechanisms relatively preserved.

Recent functional imaging studies corroborate our results suggesting that HD may impact these networks differently. On a stop signal task, prodromal HD patients far from motor onset demonstrated hyperactivation of the right SMA and anterior cingulate, which subsequently decreased with disease progression and correlated with worsening response inhibition (Rao et al., 2014). Another study showed enhanced activation of SMA and pre-SMA in pre-symptomatic HD gene carriers that appeared to play a compensatory role in maintaining executive motor control (Klöppel et al., 2009). Based on average CAP score, our subjects were in the early motor-manifest stage of the disease, and given the close association previously suggested between pre-SMA activation and susceptibility to prepotent action impulses, hyperactivation in this region may be one explanation for the high rate of fast, impulsive errors seen in our study.

To explain the preservation of impulse suppression, there is also evidence that HD patients performing the Simon task show increased activation in the anterior cingulate, prefrontal, and parietal cortex compared to controls, including significantly increased activity in the right inferior frontal gyrus (Georgiou-Karistianis et al., 2007; Thiruvady et al., 2007). Previous MRI studies implicated striatal atrophy in HD population before the onset of motor symptoms (Aylward et al., 2004; Paulsen et al., 2010). Increased recruitment of cortical areas involved in inhibitory action control may thus allow for some degree of compensation for degeneration of striatal regions. This could be clinically relevant as well, as situations that are cognitively demanding or that present with conflicting information (e.g., navigating a complex urban environment or social interactions) may require patients to deliberately slow down to avoid making impulsive errors. Our results suggest that HD patients may be able to do so proficiently, at least early in the disease process. This is an important observation that emphasizes the need for introducing targeted psychological and behavioral therapies early in the disease course and lends support to recent work showing that mindfulness-based cognitive therapy is effective for reducing psychiatric symptoms and improving emotion regulation in premanifest HD (Eccles et al., 2021).

When comparing HD patients’ performance on the Simon action control task to patients suffering from other movement disorders like Essential Tremor (ET) and Parkinson disease (PD), the pattern of increased impulsive errors and relatively intact inhibitory control seems to overlap with what has been found in ET (Kane et al., 2022), but is different from performance in PD patients (Wylie et al., 2012; van Wouwe et al., 2016b). Interestingly, dopaminergic medications in PD improve top-down inhibitory control but have no effect on impulsive error rates (van Wouwe et al., 2016b). This suggests that impulse capture may be a dopamine-independent process or that a hyperdopaminergic state can potentially trigger problems in impulse capture. The role of dopamine in HD is less clear, although alterations in striatal dopamine transmission and dysfunction of the indirect pathway have been associated with impairments in inhibitory control as well as the cognitive and psychiatric manifestations of this disease (Majid et al., 2013; Schwab et al., 2015; Rangel-Barajas and Rebec, 2016; Koch and Raymond, 2019). Further research is needed to elucidate the neurobiological implications of these findings and their relationship to cognitive and behavioral symptoms.

### Limitations

There are a few limitations that are worth mentioning for this study. First, the sample size of our study was relatively small and only included early manifest HD patients. A larger sample of participants, including premanifest and more advanced patients, would help to corroborate these results and provide further insights into inhibitory control dynamics and potential compensatory mechanisms at different stages of the disease. In terms of demographic characteristics, participants with HD had a significantly lower mean level of education than HCs. This is representative of our broader HD clinic population at Vanderbilt, in which 40% of patients have less than 12 years of formal education (Pfalzer et al., 2021). A previous study using a modified version of the Simon Task (EAST) that was more difficult to perform found that there was no influence of age or education level on the result (Huijding and De Jong, 2005). To verify this, we examined the magnitude of the Simon effect by education level and found no significant correlation for either HD patients or HCs in our sample.^1^ HD participants were also more likely to be left-handed than HCs. However, mapping of color and response hand were counterbalanced across participants, and the magnitude of the Simon effect did not differ between left- and right-handed participants, so this difference is unlikely to introduce a significant confound. Finally, we did not consider medication effects in this study. Based on our previous work in PD, both dopamine deficiency and dopamine supplementation are associated with impaired impulse suppression but no change in impulse capture. Therefore, any antidopaminergic medications used in HD could potentially worsen overall inhibitory control but would not affect the rate of fast, impulsive errors. Medication effects would therefore be unlikely to change or account for our results, but this could be explored further in future studies.

## Conclusion

The results of this study provide novel information regarding the temporal dynamics of response impulses in HD patients, showing a clear dissociation between exacerbated capture by prepotent response impulses and preserved interference suppression. The finding of stronger impulse capture may offer a novel metric or target for behavioral intervention studies aimed at reducing the deleterious effects of impulsive behavior. Further research is needed to elucidate the underlying neurobiological implications of these findings and their relationship to clinically observed behavioral symptoms.

## Data availability statement

The raw data supporting the conclusions of this article will be made available by the authors, without undue reservation.

## Ethics statement

The studies involving human participants were reviewed and approved by Vanderbilt University Medical Center Institutional Review Board. The patients/participants provided their written informed consent to participate in this study.

## Author contributions

NvW, SW, and DC contributed to the conception and design of the study. NvW and KM performed the statistical analysis. NvW created the figures. SS wrote the first draft of the manuscript and organized the database. KM and NvW wrote sections of the manuscript and edited the content. All authors contributed to the article and approved the submitted version.

## Funding

This study was supported in part by NINDS K23 NS126628 (KM).

## Conflict of interest

The authors declare that the research was conducted in the absence of any commercial or financial relationships that could be construed as a potential conflict of interest.

## Publisher’s note

All claims expressed in this article are solely those of the authors and do not necessarily represent those of their affiliated organizations, or those of the publisher, the editors and the reviewers. Any product that may be evaluated in this article, or claim that may be made by its manufacturer, is not guaranteed or endorsed by the publisher.

## Footnotes

^1^Spearman’s correlations for Simon effect and education: HD: Acc *r* = −0.018, *p* = 0.95; RT *r* = 0.22, *p* = 0.44. HC: Acc *r* = 0.08, *p* = 0.77; RT *r* = −0.39, *p* = 0.14.
